# Transcending membrane barriers: advances in membrane engineering to enhance the production capacity of microbial cell factories

**DOI:** 10.1186/s12934-024-02436-8

**Published:** 2024-05-25

**Authors:** Tao Wu, Jingjing Jiang, Hongyang Zhang, Jiazhi Liu, Haihua Ruan

**Affiliations:** Tianjin Key Laboratory of Food Science and Biotechnology, College of Biotechnology and Food Science, Tianjin University of Commerce, Tianjin, China

**Keywords:** Membrane engineering, Microbial cell factories, *Escherichia coli*, Yeast

## Abstract

Microbial cell factories serve as pivotal platforms for the production of high-value natural products, which tend to accumulate on the cell membrane due to their hydrophobic properties. However, the limited space of the cell membrane presents a bottleneck for the accumulation of these products. To enhance the production of intracellular natural products and alleviate the burden on the cell membrane caused by product accumulation, researchers have implemented various membrane engineering strategies. These strategies involve modifying the membrane components and structures of microbial cell factories to achieve efficient accumulation of target products. This review summarizes recent advances in the application of membrane engineering technologies in microbial cell factories, providing case studies involving *Escherichia coli* and yeast. Through these strategies, researchers have not only improved the tolerance of cells but also optimized intracellular storage space, significantly enhancing the production efficiency of natural products. This article aims to provide scientific evidence and references for further enhancing the efficiency of similar cell factories.

## Introduction

Microbial cell factories adopt cost-effective and environmentally friendly strategies, utilizing inexpensive renewable resources to produce a wide range of high-value compounds, including fine chemicals, biofuels, natural products, and pharmaceuticals [1–4]. With the growing demand for enhanced performance of cell factories, the academic community has undertaken extensive and in-depth research efforts. Advanced technologies such as gene editing [5–8], protein engineering [9–11], and dynamic genetic circuits [10–12] have been widely applied in the construction and performance optimization of microbial cell factories. Although traditional synthetic biology strategies focus on the construction and optimization of metabolic pathways, such as strengthening central and major metabolic pathways [13–15], improving the supply and turnover of cofactors [16, 17], removing competitive metabolic bypaths, and enhancing metabolic flow [18, 19], these methods have shown significant limitations in increasing the yield of many hydrophobic natural products (e.g., steroids, terpenoids, alkaloids, and liposoluble vitamins). In particular, many hydrophobic natural products tend to accumulate in biological membrane structures rather than being secreted into the culture medium, a tendency that, due to the limited space of the cell membrane structure, restricts the effective accumulation of target chemicals. Furthermore, excessive accumulation of these hydrophobic natural products on membrane structures may interfere with the normal physiological functions of the host cells, causing cytotoxicity, thereby worsening the robustness and productive efficiency of the strains. Therefore, engineering the membrane structure of microbial chassis cells to improve cell tolerance and optimize intracellular storage space is crucial for enhancing the capability of microbial cell factories to produce natural products. In recent years, cell membrane engineering, as a strategy for optimizing the performance of cell factories, has gradually attracted widespread attention in the academic community and has led to several successful modification cases. This article reviews the progress of membrane engineering technology research for prokaryotic and eukaryotic microbial cell factories in recent years, summarizes the advantages and limitations of different strategies, and looks forward to new membrane engineering strategies and their potential applications.

## Applications of membrane engineering in prokaryotic cell factories: the case of *Escherichia coli*

**Fig. 1:**
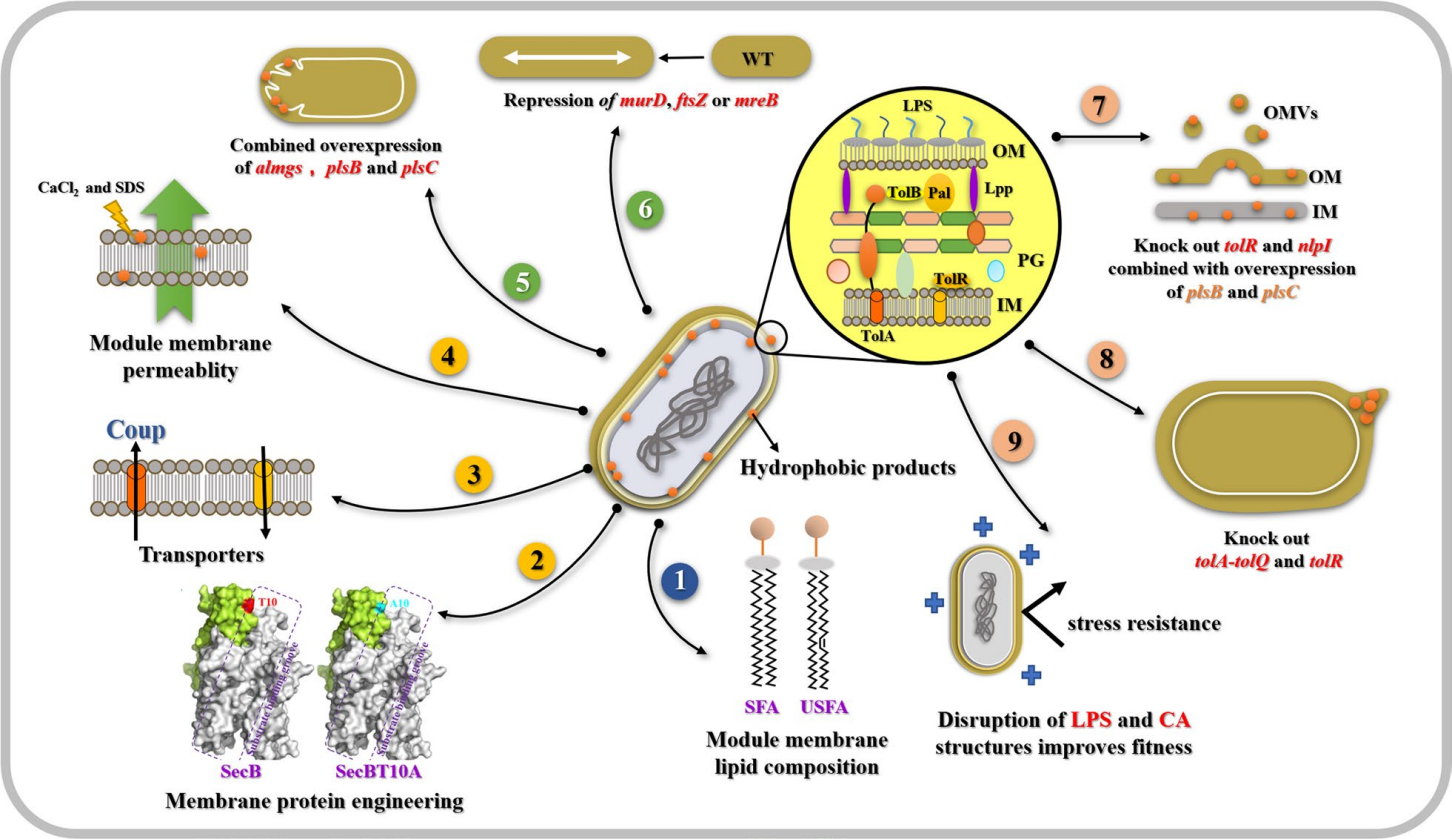
Membrane engineering strategies used in improving *E. coli* cell factories performance. Different numbers with the same colour represent the same kind strategy. ①: Module membrane lipid composition; ②③④: Membrane protein engineering; ⑤⑥:Membrane morphology engineering; ⑦⑧⑨: Outer membrane engineering

*Escherichia coli*, as a typical representative of prokaryotic cell factories and a member of the Gram-negative bacteria, possesses a cell envelope structure composed of an outer membrane, periplasmic space, and inner membrane. In its natural state, the cell membrane of *E. coli* is intact, smooth, and evenly distributed. However, according to existing literature, the morphology of the cell membrane can be altered by overexpressing certain endogenous membrane proteins in *E. coli*. Specifically, the induced expression of ATP synthase (Atp) can induce the formation of membrane cisterns and vesicle structures within the cell [20]; whereas the induction of mannitol permease (MtlA) expression can cause membrane stacking in the vicinity of the inner membrane [21]. Moreover, the induced expression of chemotaxis receptor (Tsr) and sn-glycerol-3-phosphate acyltransferase (PlsB) can promote the formation of a large number of intracellular tubular structure, effectively changing the morphology of the cell membrane [22, 23]. Research by Eriksson et al. further demonstrated that overexpressing the membrane-bound 1,2-diacylglycerol 3-glucosyltransferase from *Acholeplasma laidlawii* (AlMGS) in *E. coli* significantly increases the number of intracellular membrane vesicles, an effect far surpassing that of *E. coli*’s endogenous proteins with similar functions [24, 25]. This finding makes it possible to expand the cell membrane area. Although there is a wide variety of engineering modification strategies for bacterial chassis currently available, the productive performance of chassis strains can still be negatively affected during the fermentation process due to feedback inhibition by products and the accumulation of metabolic by-products [26, 27]. Studies have shown that the toxicity of products is a major factor leading to cell membrane rupture. Therefore, regulating and remodeling the components of the cell membrane to enhance its stability and improve the robustness of the strains has become an important hot topic in current research. The following Table 1 and Fig. 1 summarize main strategies for the engineering modification of *E. coli* membranes to enhance strain tolerance and improve the performance of cell factories.

**Table 1:** The impact of membrane engineering modifications on the performance of *E. coli* cell factories

Strain	Engineering Strategy	Result	References
*E. coli*	Adaptation to octanoic acid	Fivefold increase in octanoic acid production	[29]
*EcNR1 G3.2*	Parallel evolution with isobutanol	Isobutanol production reached 5 g/L	[30]
*E. coli* MG1655	Overexpression of *cfa* gene	Increase in cyclopropane fatty acids content and butanol tolerance	[33]
*E. coli* MG1655	Overexpression of *cti* gene	Increased production of octanoic acid	[34]
*E. coli* MG1655	Overexpression of *pssA*	Increased production of octanoic acid	[38]
*E. coli* MG1655	Combined knockout of *clsA*, *clsB*, and *clsC*	2.48-fold increase in chlorogenic acid production	[39]
*E. coli* E1-A1-thrA-serAc	Improved cell membrane permeability	Ergothioneine production of 243.06 mg/L	[43]
*E. coli* DH1	Regulation of transporter CouP expression	30–40% increase in catechol production	[41]
*E. coli* JM109	Overexpression of *SecB*_*T10A*_	5.3-fold increase in n-butanol tolerance	[45]
CAR015, CAR025	Overexpression of exogenous membrane protein AlMGS and strengthening of cell membrane synthesis pathway	39% increase in beta-carotene production per cell	[46]
LYC 101	Overexpression of *almgs*, *plsB*, and *plsC*	Increased lycopene production	[47]
LYC79, BTC1, et al	System metabolic engineering, membrane engineering, cell morphology	Increased production of astaxanthin and other pigments	[48]
*E. coli* JM109	Repression of *murD* and *murE*	93% increase in PHB production	[49]
*E. coli* JM109	Repression of *mreB* expression	71% increase in PHB production	[50]
CAR015, CAR025	AVMTS system	61% increase in beta-carotene production	[79]
LYC series	Knockout of *TolA*-*TolQ* and *TolR*	Increased lycopene production	[82]
*E. coli* W3110	Disruption of LPS structure, Colanic acid structure	Increase in PHB production	[53, 83]
*E. coli* JM 109	Disruption of cell membrane integrity	Increase in PHB production	[49]

### Membrane lipid composition engineering

By employing adaptive evolution strategies for the modification of *E. coli* strains, significant improvements can be made in their growth performance under adverse environmental conditions. Research by Royce et al. revealed that short-term metabolic evolution of *E. coli* in an octanoic acid environment can increase the ratio of unsaturated to saturated fatty acids (U/S) and the number of acyl chains, thereby reducing the hydrophobicity of the cell surface. In contrast, strains that had not undergone octanoic acid adaptive modification showed increased membrane fluidity and decreased tolerance to the product octanoic acid [28]. Another study has shown that by using evolutionary methods to increase *E. coli*’s tolerance to exogenous octanoic acid, the evolved strain LAR1, as compared to its parental strain ML115, not only acquired broader tolerance to alcohols and carboxylic acids (including octanoic acid, hexanoic acid, decanoic acid, n-butanol, and isobutanol) but also produced carboxylic acids at a titer 5 times higher than its parent [29]. The acquisition of these evolutionary traits is associated with changes in the membrane phospholipid composition; for example, LAR1 showed a significantly lower saturated:unsaturated (S:U) ratio, decreased C12/C16 and C14/C16 ratios, and an increased average membrane length relative to its parent. Furthermore, Minty et al. [30] reported that an evolved mutant strain, EcNR1 G3.2, which exhibited high fitness under exogenous isobutanol stress, contains mutations in numerous genes and regulators associated with the cell envelope. These include genes for components of the Sec apparatus such as *secA* and *lepB*, the membrane proteins regulator *hfq*, genes related to LPS biosynthesis like *fepE* and *yjgQ*, and the regulator of various LPS modification genes *phoPQ*. Compared to the wild-type, the G3.2 mutant exhibits a significant reduction in cyclopropane fatty acid content, a significant increase in the overall unsaturated/ saturated fatty acid ratio, and an overall increase in cell envelope proteins. Cyclopropane fatty acids (CFAs), as an important component of microbial fatty acids in organisms such as *E. coli*, play a crucial role in maintaining membrane lipid homeostasis and improving strain acid tolerance [31, 32]. Royce et al. through transcriptome analysis, found that under the stress of octanoic acid, isobutanol, and ethanol, the expression of the *cfa* gene, which encodes for the CFA synthase, was downregulated. Overexpression of the *cfa* gene derived from the butanol-tolerant strain *Enterococcus faecalis* in *E. coli* significantly increased the content of CFAs in the membrane, thereby improving the growth status and octanoic acid tolerance of the recombinant strain [33]. In most Gram-negative bacteria, the cytoplasmic *cis*–*trans* isomerase can convert unsaturated fatty acids from *cis* to *trans* under stress conditions, to maintain cell membrane fluidity. Tan et al. have indicated that overexpressing the *cis–trans* isomerase Cti from *Pseudomonas aeruginosa* in *E. coli* can enhance the rigidity of the cell membrane, reduce its fluidity, and thus increase *E. coli*’s tolerance and production of octanoic acid [34]. Furthermore, the expression of the *cti* gene also improves the strain’s tolerance to physical stresses such as low pH, osmotic pressure, high temperature, and to chemical stresses such as short-chain fatty acids, ethanol, organic acids, and aromatic compounds. These results indicate that modifications to the unsaturated fatty acid components in the membrane can affect its fluidity, thereby influencing chassis cells’ tolerance to bulk chemicals. Moreover, the stress tolerance of *E. coli* can be improved by adjusting the length, number, and conformation of double bonds and rings in the hydrophobic tails of fatty acids within phospholipids [35–37]. Phosphatidylserine decarboxylase plays a crucial role in the composition of cell membrane phospholipids. In the research conducted by Tan et al. [38], the overexpression of phosphatidylserine decarboxylase (pssA) resulted in an increased concentration of phosphatidylethanolamine (PE) within the cell membrane phospholipids. Given that the head group of phosphatidylethanolamine has a stronger polarity compared to that of phosphatidylglycerol (PG) and cardiolipin (CL), this led to a decrease in the hydrophobicity of the bacterial surface. Additionally, the composition of the fatty acid tails was also altered. These modifications not only enhanced the production of and tolerance to octanoic acid in *E. coli* but also increased the strain’s resistance to unfavorable conditions such as acetate, furan, toluene, ethanol, and low pH levels. Research by Wu et al. [39] demonstrated that by combining the knockout of cardiolipin synthesis-related genes *clsA*, *clsB*, and *clsC* with the enhancement of the Regulator of capsule synthesis (RCS) phosphorelay system, the production of colonic acid in *E. coli* MG1655 was increased by 2.48-fold. These results suggest that altering membrane composition components can effectively confer a strain’s tolerance to chemicals in the environment, providing a theoretical basis for the targeted development of efficient cell factories and the enhancement of production in the future.

### Membrane protein engineering

Membrane proteins comprise a range of proteins that perform various critical functions, such as transporters, receptors, enzymes, anchors, and cell adhesion molecules. The homeostasis of these proteins, to some extent, affects the homeostasis of the cell membrane. For example, transporters facilitate the movement of substances across cell membranes. Thus, introducing or enhancing the function of transporters can help reduce the accumulation of toxic products on the cell membrane, increase the robustness of chassis cells, and ultimately improve the productive capacity of cell factories. Korosh et al. [40] incorporated a lysine transport protein (encoded by the *ybjE* gene) into lysine-producing cells of *Synechococcus sp.* PCC 7002. This modification resulted in a 1.6-fold increase in the yield per unit biomass compared to the original strain and prevented intracellular lysine accumulation, which is associated with reduced fitness. Wu et al. [41] utilized an autoregulatory system in *E. coli* chassis to regulate the expression of an active aromatic transporter CouP, enabling it to efficiently transport vanillin across the cell membrane and convert it to catechol. This modification resulted in a 30–40% increase in catechol production compared to the starting strain. Additionally, modulating membrane protein homeostasis through specific reagents to affect membrane permeability is also one of the strategies for increasing production in cell factories. For example, Ca^2+^ within a physiological concentration range is sufficient to induce clustering of membrane proteins in the plasma membrane through electrostatic interactions [42], and sodium dodecyl sulfate (SDS) acts as a very effective solubilizing agent for a wide range of polypeptides, including membrane proteins. In one case, Chen et al. [43] enhanced cell membrane permeability by adding 0.2 g/dL of CaCl_2_ and surfactants such as SDS to the culture medium, combined with strategies to boost the supply of key precursors like histidine and methionine, successfully increasing the capacity of *E. coli* to synthesize unusual sulfur-containing amino acid ergothioneine, reaching a production titer of 243.06 mg/L. Furthermore, regulating the transport of membrane proteins can profoundly affect their homeostasis, which can then be utilized to enhance the production capacity of cell factories. For example, the *E. coli* soluble protein SecB has been reported involved in the export of periplasmic and outer membrane proteins, and purified SecB has been shown to stimulate protein translocation across *E. coli* inner membrane vesicles in vitro [44]. Xu et al. [45] utilized protein engineering to create 2800 random mutants of the SecB protein and identified the SecB_T10A_ mutant as having the highest tolerance to n-butanol. This modification led to a 5.3-fold increase in n-butanol tolerance in the *E. coli* chassis, resulting in n-butanol production reaching 14.58 g/L.

### Membrane morphology engineering

Increasing the surface area of the cell membrane is the most direct way to enhance the carrying capacity for hydrophobic compounds. In our prior research, the heterologous expression of AlMGS protein led to an observed increase in cell membrane surface wrinkling [46, 47]. This not only enlarged the surface area of the cell membrane but also provided extra storage space for products such as β-carotene and lycopene, significantly boosting their production levels. Additionally, our study examined the effects of enhancing the cell membrane synthesis pathway on the morphology of carotenoid-producing chassis cells and their capacity for intracellular storage. We found that enhancing the expression of proteins plsB and plsC, which are directly involved in the synthesis of phosphatidylethanolamine, promoted cell membrane synthesis, markedly increasing product yield compared to the initial strain CAR015. By integrating these strategies, a synergistic effect was found in enhancing cell factory performance. Implementing these strategies in the β-carotenoid high-producing strain CAR025 under shake-flask fermentation conditions led to a 39% increase in per unit biomass yield, a strategy applicable to lycopene cell factories as well. Moreover, Yang et al. [48] have reported the implementation of integrated membrane engineering strategies to augment the production of three types of carotenoids and four derivatives of violacein. They modified cell morphology by silencing genes involved in cell division or cell wall metabolism, thereby enlarging the membrane surface area to enhance the accumulation space for hydrophobic products. After fermentation optimization, production of rainbow colorants are significantly enhanced to 322 mg/L of astaxanthin (red), 343 mg/L of β-carotene (orange), 218 mg/L of zeaxanthin (yellow), 1.42 g/L of proviolacein (green), 0.844 g/L of prodeoxyviolacein (blue), 6.19 g/L of violacein (navy), and 11.26 g/L of deoxyviolacein (purple). Additionally, reshaping the morphology of *E. coli* has also been successfully used to improve the performance of poly-3-hydroxybutyrate (PHB) cell factories. To expand the internal space of cells and mitigate the effects of certain inhibitors on the cell wall, Zhang et al. [49] inhibited enzymes related to cell wall synthesis, such as murD and murE. This flexibility in cell shape increased the possibility for cell size expansion, providing more space for PHB storage, with PHB accumulation reaching 93% of the cell dry weight. Furthermore, to increase cell volume, Elhadi et al. [50] employed CRISPRi technology to suppress the expression of the tubulin protein *ftsZ* and the cytoskeletal protein *mreB*, which are associated with cell differentiation and peptidoglycan synthesis, to balance cell growth and morphology regulation. Ultimately, depending on the level of suppression of *ftsZ* and *mreB*, an increase in cell volume was observed to various extents. The PHB production by the mutant increased by 71% compared to the control.

### Out membrane and associated vesicles engineering

The outer membrane of *E. coli* consists of various components, including peptidoglycan (PG), lipopolysaccharides (LPS), colanic acid (CA), and outer membrane proteins (Omps), among others. Lipopolysaccharides play a dominant role in the outer layer of the outer membrane in Gram-negative bacteria and are crucial for the formation of other molecules on the outer membrane [51, 52], including flagella, CA, and Omps [53, 54]. It is estimated that each *E. coli* cell contains approximately 10^6^ LPS molecules and 10^7^ glycerophospholipid molecules [51, 55]. The vast majority of life forms, including eukaryotes, archaea, and bacteria, are capable of producing membrane-bound cellular structures, such as membrane vesicles, microvesicles, exosomes, and virus-like particles [56]. During normal growth, most Gram-negative bacteria naturally secrete Outer Membrane Vesicles (OMVs), which are nanoscale structures ranging from 20 to 250 nm in diameter, formed by the shedding from the cell wall and outward bulging of the outer membrane. Although the exact mechanisms of OMV formation are not fully understood, it is hypothesized that they may arise through three main potential mechanisms: (1) weakened cross-linking interactions between the peptidoglycan layer and the outer membrane; (2) accumulation of peptidoglycan fragments and misfolded proteins in the cytoplasm exerting pressure on the outer membrane, prompting membrane budding and vesicle formation; (3) enrichment of membrane curvature-inducing molecules such as B-band lipopolysaccharide and *Pseudomonas* quinolone signal (PQS) [57–60]. Typically, signals inducing OMV formation are closely related to environmental stress encountered by the bacteria, such as temperature stress, amino acid deprivation, phage or antibiotic attack [61, 62]. Additionally, certain culture medium components can also promote OMV production; for instance, hexadecane can induce the production of OMVs in *Acinetobacter*, while *Yersinia spp.* can produce outer membrane vesicles under glucose stimulation [63, 64]. OMV formation is also related to the cell’s growth cycle, with organisms like *Neisseria meningitidis* and *Francisella* primarily secreting OMVs during the late growth phase, whereas *Borrelia burgdorferi* and *Gallibacterium anatis* secrete OMVs during the logarithmic growth phase, and Legionella pneumophila can produce OMVs at various stages of cell growth [65–67]. Thus, Gram-negative bacteria can continuously produce OMVs at all stages of growth, and external stimuli can enhance OMV production. Naturally formed OMVs play significant physiological roles, such as reducing the intracellular concentration of organic toxins like toluene, enhancing microbial survival in harsh environments [68]; neutralizing antimicrobial peptides [69]; aiding phage release [70, 71]; removing substances that could burden the cell (such as unfolded cytoplasmic proteins); and facilitating the nucleation process during biofilm formation [72, 73]. Moreover, outer membrane vesicles released from the cell walls of pathogenic bacteria play a key role in the interaction between host and pathogen, including mediating the entry of pathogenic components into host cells and modulating the host’s defense and response capabilities [74–76]. As crucial components of the biofilm, OMVs play a vital role in intercellular communication and nutrient acquisition [77, 78].

Endogenous vesicle transport systems play a critical role in improving the transport process of hydrophobic compounds from organelles to the plasma membrane. In recent years, the academic community has delved into strategies for constructing artificial transport systems aimed at facilitating the extracellular transport of intracellular products, thereby indirectly enhancing the continuous production of these products while mitigating adverse effects on the cells. Our previous research focused on increasing the production of carotenoid-producing *E. coli* by targeting key genes involved in the formation of Outer Membrane Vesicles (OMVs), including those in the Tol-Pal complex (TolR, TolA), affecting the expression and localization of outer membrane proteins (nlpI), and an outer membrane protein (OmpF), to construct an Artificial Membrane Vesicle Transport System (AMVTS) [79]. Our findings indicated that the individual knockout of either *tolR* or *nlpI* genes significantly increased β-carotene production. Simultaneous knockout of *tolR* and *nlpI* resulted in a 35.6% increase in β-carotene production compared to the starting strain. This may be due to the disruption of these genes affecting the facilitation or integrity of the outer membrane, enhancing the formation of OMVs, and thus enabling the transport of more products through the formed OMVs. Additionally, the work by Yang et al. [48] on enhancing the production of rainbow colorants also employed vesicle engineering strategies. They augmented the generation of inner-membrane vesicles (IMVs) and outer-membrane vesicles (OMVs) within the *E. coli* chassis cells. The production of these vesicular structures was facilitated by the introduction of the *cav1* gene, encoding human caveolin-1 for IMV formation, and the silencing of genes implicated in the formation of OMVs, respectively. Phospholipids, as major components of the outer membrane, including 5% cardiolipin (CL), 70%-80% phosphatidylethanolamine (PE), and 20–25% phosphatidylglycerol (PG), play a crucial role in maintaining the normal function of the outer membrane [80, 81]. In our previous AMVTS research, we also introduced a phospholipid synthesis module to enhance membrane phospholipid synthesis in strains with *tolR* and *nlpI* knocked out, thereby exploring its impact on the strain’s efflux capability. The results showed that the carotenoid artificial transport system constructed significantly improved the transport capability of carotenoids in carotenoid-producing chassis, further promoting the intracellular production of carotenoids. This strategy resulted in a 61% increase in yield for CAR025. Similarly, Fordjour et al. [82], by knocking out genes such as TolA-TolQ TolR, successfully constructed a vesicle system in *E. coli*, thereby improving cell membrane permeability and increasing lycopene production. In application cases aimed at enhancing the PHB production of cell factories, Wang et al. through genomic and transcriptomic analysis, studied flagella assembly in the wild-type *E. coli* W3110 strain and mutant strains DwaaF, DwaaC, and DwaaG, which only synthesize lipopolysaccharides of varying lengths, concluding that flagella assembly in *E. coli* depends on the length of lipopolysaccharides; by disrupting all gene clusters related to the polysaccharide part of lipopolysaccharides (LPS), colanic acid (CA), flagella, and/or fimbriae, the outer membrane of *E. coli* W3110 was successfully modified, creating favorable conditions for PHB production [53, 83]. In addition, Zhang et al. found that disrupting membrane integrity or altering the rigidity of the outer membrane could significantly enhance the PHB production yield in *E. coli* JM109 [49]. In summary, targeted modifications of the outer membrane of *Escherichia coli* and the development of artificial vesicle transport systems have proven to be effective strategies for enhancing the production of specific hydrophobic products in recent years, broadening the approach to the development of synthetic biology tools.

## Applications of membrane engineering in eukaryotic cell factories: the case of yeast hosts

**Fig. 2:**
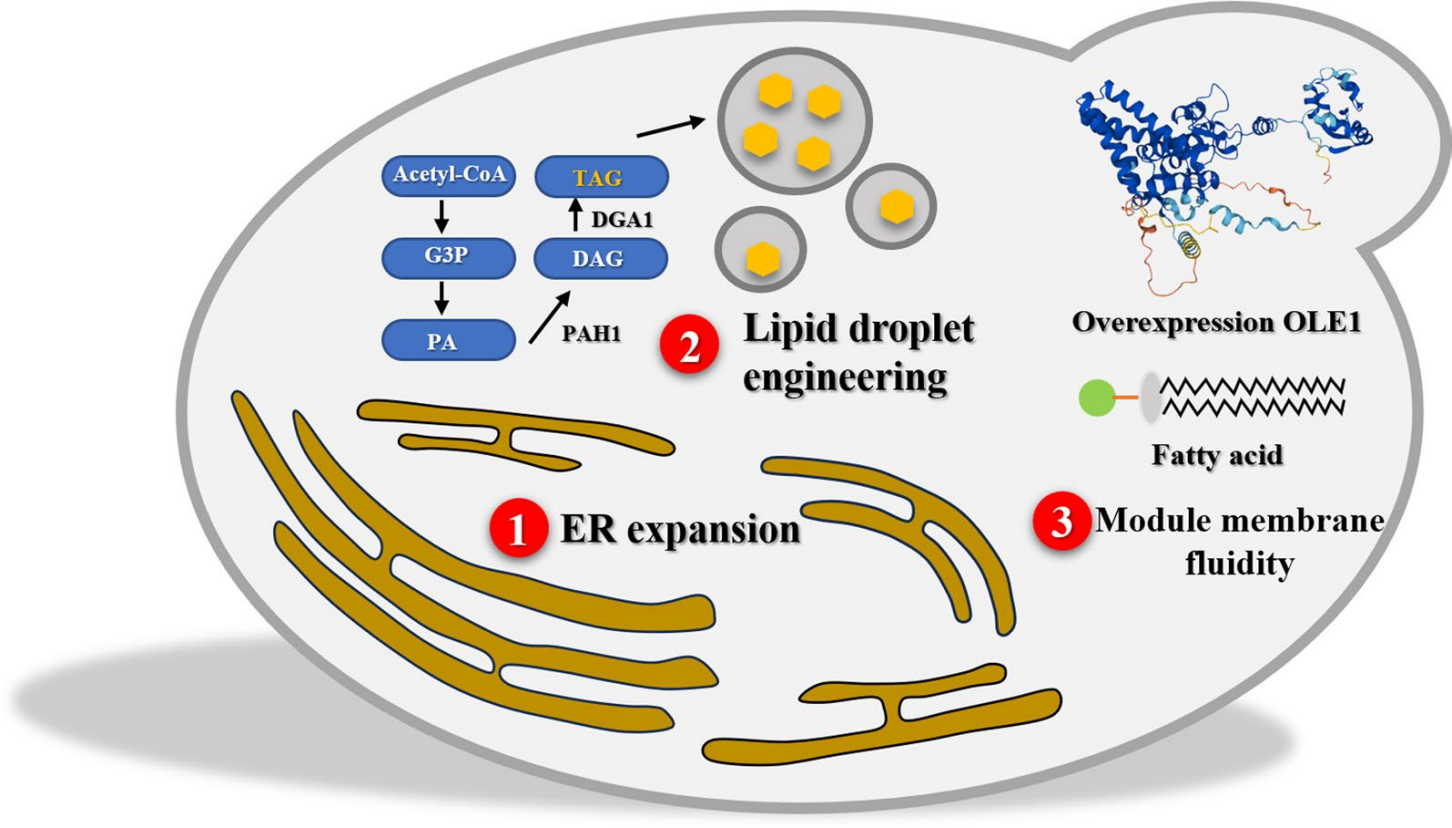
Membrane engineering strategies mainly used in improving yeast cell factories. ①: Endoplasmic reticulum (ER) expansion; ②: Lipid droplet (LDs) engineering; ③: Membrane fluidity modulation.

Eukaryotic cells show significant distinctions from prokaryotic cells in membrane phospholipid composition. The complexity of eukaryotic cell membranes is higher, featuring the evolution of steroid molecules, viewed as an adaptation to rising levels of oxygen in Earth’s atmosphere [84]. Yeast cells, for example, manage their membrane fluidity and stability by producing ergosterol, indicating fungi’s evolutionary adjustment to environmental shifts [85]. Eukaryotic cell membranes consist of a broad array of glycerophospholipids, including phosphatidylcholine, phosphatidylethanolamine, phosphatidylserine, phosphatidylinositol, and phosphatidic acid. Phosphatidylcholine, making up more than 50% of the phospholipids in most eukaryotic cell membranes, underscores its crucial role in membrane structure and functionality [86]. Moreover, eukaryotes have a sophisticated internal membrane system, encompassing organelles with internal membranes such as mitochondria, endoplasmic reticulum, and Golgi apparatus, with the endoplasmic reticulum serving as the main site for lipid synthesis [87]. The mammalian Golgi apparatus not only synthesizes sphingolipids but also contributes to the production of complex lipids like sphingomyelin (SM), glucosylceramide (GlcCer), lactosylceramide (LacCer), and more structured glycosphingolipids [88]. The asymmetric distribution and transfer of lipids, causing lipid imbalance, are vital for membrane curvature induction and vesicle budding, crucial for packaging and transporting cellular materials [67, 89]. Diverse membrane systems in eukaryotes showcase variation in composition and transport mechanisms. Even within the same type of organelle, compositional variances can be observed across different eukaryotic levels, for instance, in cardiolipin content between mammalian and yeast mitochondria. Such differences in organelle membrane architecture significantly impact the synthesis and storage efficiency of heterologous natural products in microbial systems, mirroring eukaryotic cells’ evolutionary responses and adaptations to environmental challenges. Table 2 and Fig. 2 outlines strategies for enhancing cell factory performance via yeast membrane engineering alterations.

**Table 2:** The impact of membrane engineering modifications on the performance of yeast cell factories

Strain	Engineering strategy	Result	References
*S. cerevisiae* W303-1A	Overexpression of FAD2	Enhanced tolerance to salt stress	[90]
*S. cerevisiae*	Overexpression of ELO2, enhancing sphingolipid synthesis	Enhanced cell tolerance	[91]
*S. cerevisiae* YBX-20	Overexpression of OLE1	Extracellular secretion of β-carotene and intracellular β-carotene production increased by 5.80 and 1.71 times compared to YBX-01	[92]
*S. cerevisiae* JHY-874D7	Overexpression of OLE1, increasing UFA content	Lycopene production increased by 74.6 times	[93]
*S. cerevisiae* CEN.PK2	Upregulation of factors like inO2	Lycopene production increased by 22 times	[28]
*Y. lipolytica*	Enhanced lipid droplet synthesis, overexpression of OLE1	Lycopene content reached 70.5 mg/g DCW	[94]
*S. cerevisiae* YBX	Enhanced lipid droplet synthesis, overexpression of ERG9	Per-cell β-carotene production increased by 107.3%	[97]
*S. cerevisiae*	Co-localization of Atf1 with lipid droplets	Ethyl acetate production doubled	[98]
*S. cerevisiae*	Co-localization of PPDS with lipid droplets	Conversion rate of dammarenediol II increased by 3.9 times	[99]
*S. cerevisiae*	Expansion of the endoplasmic reticulum	Squalene content increased by 71 times	[100]
*S. cerevisiae*	Knockout of phosphatidic acid phosphatase PAH1, overexpression of Ino2p	Soyasaponin production increased by 20%	[101]
*S. cerevisiae*	Knock out *PAH1* gene	β-amyrin, medicagenic acid, medicagenic-28-o-glucoside increased 8,6 and 16 times compared with the original strain	[102]
*Y. lipolytica*	Overexpress DHCR7 and POX2	Campesterol content reached 942 mg/L	[103]
*Y. lipolytica*	Knockout ERG5 and overexpress DHCR 7	3.7-fold increase in campesterol production	[104]
*Y. lipolytica*	Interference with intracellular lipid synthesis, modulation of cell morphology	Increased production of taxol	[105]

### Cell membrane engineering

Rodríguez et al. [90] expressed two sunflower (*Helianthus annuus*) oleic acid delta-12 desaturases, encoded by *FAD2-1* and *FAD2-3*, in the wild-type yeast strain W303-1A (*trp1*) and analyzed their effects on growth and stress tolerance. The result showed that by adjusting the concentration of monounsaturated fatty acids in *Saccharomyces cerevisiae* and converting them to dienoic acids, it is possible to synergistically enhance the fluidity of the cell membrane, thereby improving the yeast strain’s tolerance to freezing stress and NaCl stress. The engineered strain overexpressing FAD2 showed a survival rate of 90% after 35 days at 20 °C, compared to only 50% for the wild-type strain. Zhu et al. [91] revealed that sphingolipid acyl chain elongase (ELO2) is related to osmotic tolerance. Overexpression of ELO2 in *Saccharomyces cerevisiae* increases the content of long-chain fatty acids in sphingolipid, thereby enhancing the integrity of cell membrane and the cell’s tolerance to osmotic pressure. Addressing the problem of decreased membrane fluidity and insufficient secretion efficiency caused by β-carotene, Bu and others intervened by overexpressing the stearoyl-CoA desaturase (OLE1) [92]. Under these conditions, cell membrane fluidity was enhanced, and the secretion capacity for β-carotene was improved. Compared to the starting strain YBX-01, the extracellular secretion and intracellular production of β-carotene by strain YBX-20 increased by 5.80 and 1.71 times, respectively. Similarly, to alleviate the cytotoxicity of lycopene, Hong et al. [93] increased the content of unsaturated fatty acids in the cell membrane and enhanced cell protection and product yield by overexpressing the stearoyl-CoA desaturase encoded by *OLE1* and the transcription factor encoded by *STB5* involved in NADPH generation. Compared to the starting strain, the production of lycopene was successfully increased by 74.6 times. Chen et al. [27] by modifying the membrane composition of *S. cerevisiae*, including regulating stress response factors and synthesizing sterols and phospholipids-related factors (such as INO2) to enhance lycopene production, increased the lycopene yield of the engineered strain by 22 times compared to the starting strain.

### Lipid droplet engineering

Lipid droplets (LDs) are spherical organelles surrounded by a monolayer of phospholipids, with over 200 different structural and functional proteins localized on their surface, such as acyl-CoA diacylglycerol acyltransferase (DGAT) and Perilipin, and a core of neutral lipids like triacylglycerols (TAGs), which are stored in lipid droplets mainly in the form of lipid esters. LDs serve as storage sites for lipophilic compounds. Matthäus et al. [94] found that by enhancing the formation of LDs in *Y. lipolytica* and overexpressing the rate-limiting genes for isoprenoid biosynthesis, GGS1 and HMG1, the cell’s capacity to store lycopene was improved, subsequently increasing lycopene synthesis with a yield reaching 16 mg/g of dry cell weight (DCW). Similarly, by overexpressing diacylglycerol acyltransferase (DGA1) in *S. cerevisiae* to increase its intracellular storage capacity and enhancing the expression of the key mevalonate (MVA) pathway gene acetyl-CoA carboxylase (ACC1), the engineered strain’s fermentation yield of α-amyrin was increased by 106 times compared to the wild-type strain [95]. Additionally, by overexpressing the fatty acid desaturase (OLE1) in *S. cerevisiae* and knocking out the seipin gene (FLD1), which regulates LDs size, lycopene production reached 73.3 mg/g DCW [96]. Xia et al. [97], by regulating the expression of triglycerides in LDs and the ERG9 protein, achieved yeast cell β-carotene production of 11.4 mg/g DCW and 142 mg/L, which was 107.3% and 49.5% higher than the original strain, respectively, thereby alleviating the stress on the cell membrane caused by β-carotene accumulation. Lin et al. [98] developed a protein scaffold system to organize and bring together multiple enzymes involved in yeast ester biosynthesis on the surface of intracellular LDs. This setup mimics the cell’s natural organization, aiming to improve metabolic pathways that can become inefficient when such organization is lost. By using the plant protein oleosin and cohesin-dockerin interaction pairs, they managed to gather the enzymes near the location of the key enzyme, alcohol-o-acetyltransferase (Atf1), which is crucial for the process. This close arrangement of enzymes boosted the efficiency of the ester biosynthesis pathway. By testing different scaffold designs and levels of pathway expression, they found a setup that doubled the production rate of ethyl acetate compared to the unorganized pathway. This approach shows promise for optimizing other metabolic pathways, especially those involving reactions on lipid droplets or membranes. Shi et al. [99] co-localized protopanaxadiol synthase (PPDS), involved in ginsenoside biosynthesis, with its substrate dammarenediol II in LDs, enhancing the conversion rate of dammarenediol II to protopanaxadiol in yeast by 3.9 times to 86%.

### Intracellular membrane engineering

Due to the imbalance between the protein synthesis load on the endoplasmic reticulum (ER) and its folding capacity triggering the unfolded protein response, normalization of ER function necessitates size adjustments. In the research conducted by Kim et al. [100], a significant increase in squalene production was achieved, reaching 634 mg/L, which was a 71-fold increase compared to the control group, by combining ER expansion with other metabolic engineering strategies. Liu et al. [101] expanded the ER in *S. cerevisiae* by knocking out the phosphatidic acid phosphatase *PAH1* gene and overexpressing the transcription factor INO2, thereby increasing the production of soyasaponin by 20% compared to the control strain. Similarly, Arendt et al. [102] knocked out *PAH1* gene in *S. cerevisiae* using CRISPR-Cas9, the ER membrane was expanded and the production of β-amyrin, medicagenic acid, and medicagenic-28-o-glucoside was increased 8 times, 6 times and 16 times compared with the original strain, respectively. In the work of Zhang et al. [103], the DHCR7 from Danio rerio and POX2 were overexpressed in *Y. lipolytica* for building planticized membrane, resulting the production of campesterol to 942 mg/L in 5L bioreactor. Similarly, Qian et al.[104] deleted ERG5 and overexpressed of two copies of dhcr7 in *Y. lipolytica*, the campesterol content reached 837 mg/L, which was 3.7-fold compared with the initial strain. Furthermore, Guerfal et al. [105] increased the production of intracellular membranes and the expression of membrane proteins in yeast by interfering with key enzymatic steps in lipid synthesis; by modulating lipid synthesis and cell morphology, the production of the triterpenoid compound lupeol in *Y. lipolytica* was optimized, significantly enhancing yield through this strategy.

## Conclusion and future perspectives

Against the backdrop of rapid developments in synthetic biology and systems biology, microbial cell factories have shown immense application potential in producing high-value hydrophobic compounds. Membrane engineering, as a key technology, aims to enhance the production capacity and tolerance of cells towards these compounds by finely regulating microbial membrane structure and function, and has achieved significant results in optimizing the performance of microbial cell factories. Various engineering tools, mainly including genome editing (CRISPR-Cas9, CRISPRi) [49, 50, 101], adaptive laboratory evolution (ALE) [29, 33, 90, 91], pathway engineering (gene fine-tuning, multiple genomic integrations) [27, 96, 103] have been employed to achieve the goals of membrane engineering. The application of transcending membrane barriers is manifested not only in enhancing strain tolerance and robustness to improve the production efficiency of cell factories but also involves optimizing the accumulation and storage of target products on cell membranes to alleviate the cellular burden and stress caused by excessive accumulation.

However, although current research has demonstrated that strategies such as modifying cell membrane protein expression, adjusting lipid composition, and reshaping membrane structure can significantly enhance the efficiency of product production, these modification strategies still face numerous challenges in practical applications. Firstly, the biology of cell membranes is extremely complex, and minor changes in membrane composition can have profound effects on the cell’s overall function and stability. Thus, how to precisely regulate the modification of cell membranes to achieve a balance between increasing the production of hydrophobic compounds without disturbing the cell’s normal physiological functions represents a significant challenge in current research. Additionally, different hydrophobic compounds have varying effects on cell membranes, requiring personalized membrane engineering designs and optimizations for each target product, increasing the complexity and difficulty of research. Secondly, although membrane engineering modifications have improved microbial cell tolerance to hydrophobic compounds, efficient secretion of the products remains a challenge. For many hydrophobic compounds, their production efficiency is limited due to the toxicity caused by accumulation within the cell and the inefficiency of traditional secretion pathways. Therefore, designing new efficient secretion systems to facilitate the transport of hydrophobic compounds from the cell to the external environment is a key direction for the future development of membrane engineering. Moreover, scaling up from laboratory to industrial-scale production poses another significant challenge. Membrane engineering strategies validated under laboratory conditions are often difficult to apply directly to large-scale production processes. This is not only because the physical and chemical conditions during scaling up may affect the production performance of the modified cells but also because factors such as cost-effectiveness, production stability, and environmental sustainability need to be considered in large-scale production processes. Therefore, successfully translating membrane engineering techniques from the laboratory to industrial applications requires further research and innovation.

**Fig. 3:**
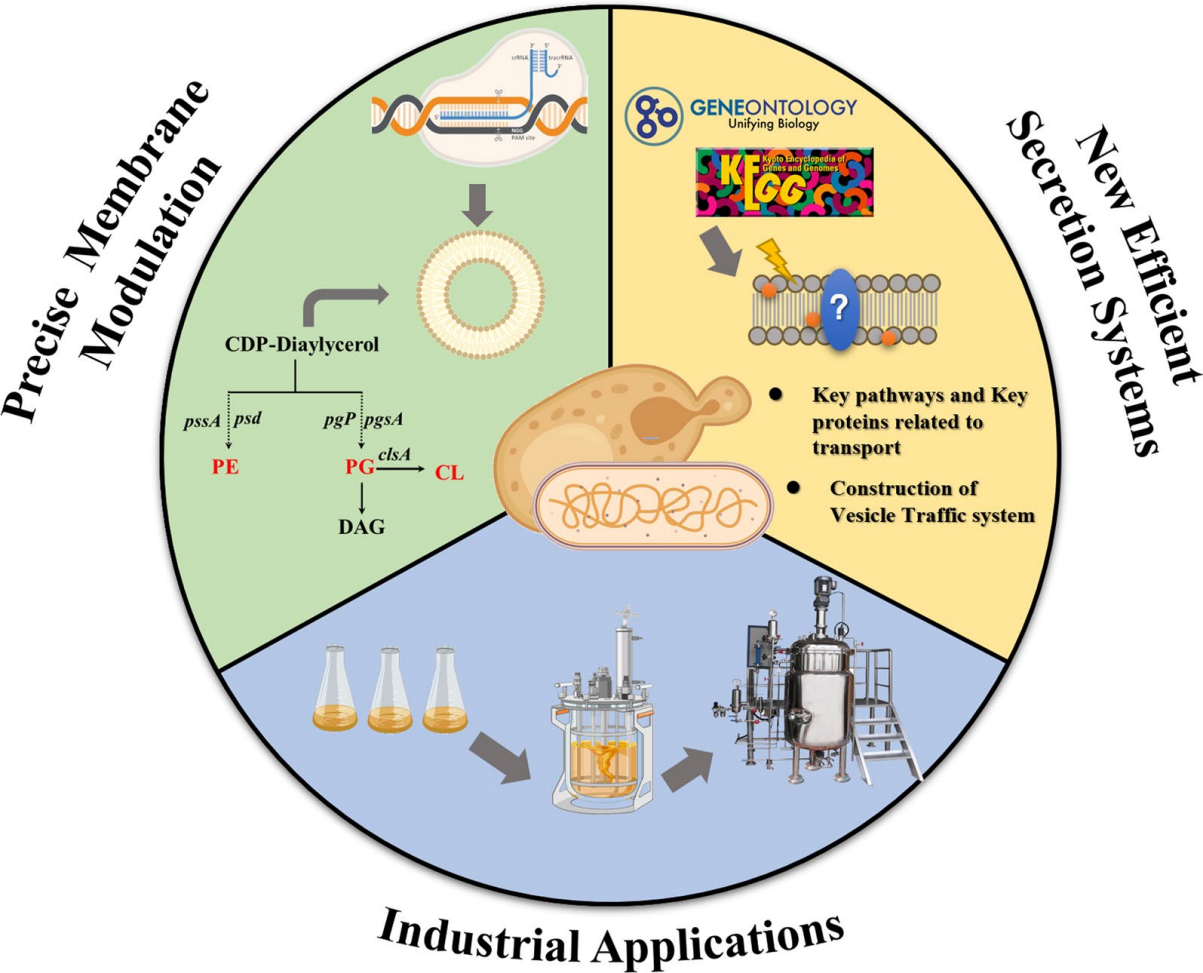
Perspective of membrane engineering

Facing these challenges, future research needs to delve into multiple areas (Fig. 3). On one hand, building on previous studies on regulating cell membrane synthesis pathways for the accumulation and storage of hydrophobic compounds like carotenoids, it is deemed necessary to conduct more comprehensive and in-depth studies on cell membrane components, especially the synthesis pathways of central phospholipids and phosphatidylglycerol. By more detailed single-gene or multi-gene regulation of different cell membrane component synthesis pathways and establishing the connection between key genes regulating membrane component synthesis pathways and the production of hydrophobic compounds, it might reveal mechanisms of hydrophobic compound accumulation on cell membranes and identify potentially relevant structures associated with cellular synthesis pathways capable of storing hydrophobic compounds, thereby finding more effective methods to improve the intracellular production, accumulation, and storage of hydrophobic compounds. On the other hand, developing new efficient secretion systems to facilitate the effective secretion of hydrophobic compounds will be key to improving production efficiency. Additionally, given the differences in cell membrane components and their proportions between prokaryotic and eukaryotic cells, which may affect the efficacy of the same compound in two types of cell factories differently, it is particularly important to deeply study the cell membrane synthesis pathways of prokaryotic and eukaryotic chassis cells, combined with a detailed analysis and discussion on the structural characteristics and synthetic properties of target compounds.

In summary, although the application of membrane engineering in microbial cell factories faces challenges, with the continuous advancement of science and technology and the deepening of interdisciplinary collaboration, its prospects in the field of biomanufacturing remain very broad. Through in-depth research and technological innovation, the future will enable more efficient, stable, and environmentally friendly biological production of hydrophobic natural products, making a greater contribution to the sustainable development of human society.

## Data Availability

No datasets were generated or analysed during the current study.
